# Bioenergetics Failure and Oxidative Stress in Brain Stem Mediates Cardiovascular Collapse Associated with Fatal Methamphetamine Intoxication

**DOI:** 10.1371/journal.pone.0030589

**Published:** 2012-01-19

**Authors:** Faith C. H. Li, Jiin-Cherng Yen, Samuel H. H. Chan, Alice Y. W. Chang

**Affiliations:** 1 Institute of Pharmacology, National Yang Ming University, Taipei, Taiwan, Republic of China; 2 Center for Translational Research in Biomedical Sciences, Kaohsiung Chang Gung Memorial Hospital, Kaohsiung, Taiwan, Republic of China; University of Pecs Medical School, Hungary

## Abstract

**Background:**

Whereas sudden death, most often associated with cardiovascular collapse, occurs in abusers of the psychostimulant methamphetamine (METH), the underlying mechanism is much less understood. The demonstration that successful resuscitation of an arrested heart depends on maintained functionality of the rostral ventrolateral medulla (RVLM), which is responsible for the maintenance of stable blood pressure, suggests that failure of brain stem cardiovascular regulation, rather than the heart, holds the key to cardiovascular collapse. We tested the hypothesis that cessation of brain stem cardiovascular regulation because of a loss of functionality in RVLM mediated by bioenergetics failure and oxidative stress underlies the cardiovascular collapse elicited by lethal doses of METH.

**Methodology/Principal Findings:**

Survival rate, cardiovascular responses and biochemical or morphological changes in RVLM induced by intravenous administration of METH in Sprague-Dawley rats were investigated. High doses of METH induced significant mortality within 20 min that paralleled concomitant the collapse of arterial pressure or heart rate and loss of functionality in RVLM. There were concurrent increases in the concentration of METH in serum and ventrolateral medulla, along with tissue anoxia, cessation of microvascular perfusion and necrotic cell death in RVLM. Furthermore, mitochondrial respiratory chain enzyme activity or electron transport capacity and ATP production in RVLM were reduced, and mitochondria-derived superoxide anion level was augmented. All those detrimental physiological and biochemical events were reversed on microinjection into RVLM of a mobile electron carrier in the mitochondrial respiratory chain, coenzyme Q10; a mitochondria-targeted antioxidant and superoxide anion scavenger, Mito-TEMPO; or an oxidative stress-induced necrotic cell death inhibitor, IM-54.

**Conclusion:**

We conclude that sustained anoxia and cessation of local blood flow that leads to bioenergetics failure and oxidative stress because of mitochondrial dysfunction, leading to acute necrotic cell death in RVLM underpins cardiovascular collapse elicited by lethal doses of METH.

## Introduction

The psychostimulant methamphetamine (N-methyl-1-phenylpropan-2-amine; METH) is a cationic lipophilic molecule with potent actions on the central and sympathetic nervous system, and affects neurochemical mechanisms responsible for regulating attention, mood and emotional responses associated with alertness or alarm conditions, body temperature, blood pressure, heart rate and appetite [1]. Because it heightens alertness and energy, induces euphoria, enhances self-respect and increases sexual pleasure, METH possesses high potential for abuse and addiction and has become a serious societal problem worldwide [2], [3]. At issue is that METH abusers have to constantly increase dosing to sustain an elevated mood and libido and a decrease in appetite and fatigue. As such, METH intoxication is a common cause of death within the abuse population [4], [5]. Worse still, continuous use at larger doses results in not only acute METH poisoning but also METH-induced sudden death [6]–[8].

Death from METH abuse has been associated with cardiovascular collapse, cerebral edema and diffuse petechial hemorrhage in brain [6], [9]. In particular, severe hypotension and bradycardia are critical omens in patients who exhibit acute METH intoxication [7], [9]. Because of the irreversible cardiovascular failure that rapidly leads to death, treatment for METH intoxication is generally difficult [8], with 100% mortality despite intensive care in a hospital setting [9]. The prevalence of METH-induced cardiovascular collapse that leads to death notwithstanding, the underlying mechanism is much less understood.

Three important clues on delineating the mechanisms that may underlie METH-induced cardiovascular collapse arise from studies by our laboratory and others on brain death. The first clue comes from the prognosis that asystole invariably takes place, after a time lag, on diagnosis of brain death [10]. This suggests that permanent impairment of the brain stem cardiovascular regulatory machinery should precede the inevitable death [11]. Another crucial clue arises from the identification by our laboratory of a common prognostic determinant among comatose patients [11] who succumbed to systemic inflammatory response syndrome, severe brain injury or organophosphate poisoning. A dramatic reduction or loss of the power density of the low-frequency (LF) component (0.04–0.15 Hz in human) in the power spectrum of arterial pressure signals consistently takes place before significant hypotension and the eventual asystole occur. We also demonstrated [12] that the origin of this life-and-death signal resides in the rostral ventrolateral medulla (RVLM), which is long known to be responsible for the maintenance of sympathetic vasomotor tone and stable blood pressure [13]. The third clue comes from our demonstration that successful resuscitation of an arrested heart depends on maintained functionality of RVLM [14], suggesting that failure of brain stem cardiovascular regulation, rather than the heart, holds the key to cardiovascular collapse.

Cerebrovascular pathology, most commonly hypoxia and hemorrhage, is present in 20% of cases in METH-elicited death [4], [15]. Of particular interests is that our laboratory found previously in experimental brain death models that the degree of tissue hypoxia in RVLM is a crucial determinant of the severity of central circulatory regulatory dysfunction [14], [16]. An immediate corollary that arises, which forms the hypothesis for the present study, is that cessation of brain stem cardiovascular regulation that resembles that in brain death because of the loss of functionality in RVLM may underlie the cardiovascular collapse elicited by METH. This hypothesis was validated in the present study. We further demonstrated that sustained anoxia and cessation of local blood flow, followed by acute bioenergetic failure because of mitochondrial dysfunction that leads to necrotic cell death in RVLM underlie METH-induced shutdown of central cardiovascular regulation that precedes cardiovascular collapse.

## Results

### Lethal doses of METH induce cardiovascular collapse

**Figure 1:**
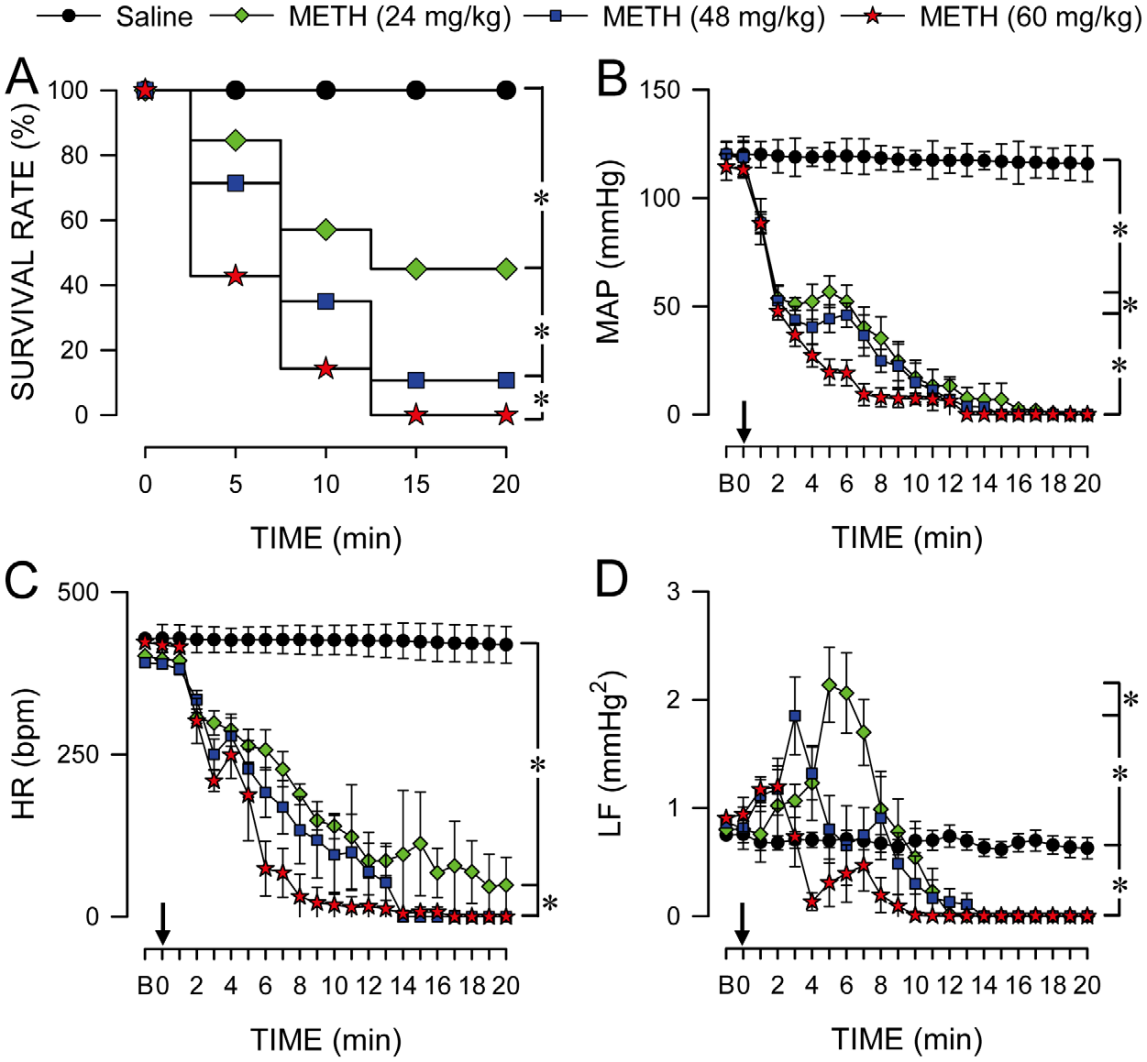
Methamphetamine dose-dependently and time-dependently elicited mortality and cardiovascular collapse. Temporal changes in survival rate (A), mean arterial pressure (MAP; B), heart rate (HR; C) or power density of the low-frequency (LF) component of systolic blood pressure signals (D) in rats that received (at arrow) intravenous (i.v.) METH or saline (vehicle control). Values are mean ± SEM, n = 5–62 animals per experimental group. *P<0.05 versus saline group in the Fisher Exact Test (A), or at corresponding time-points in the post hoc Scheffé multiple-range test (C–D). B, baseline.

Intravenous administration of METH (24, 48 or 60 mg/kg) elicited a dose-dependent decrease in survival rate (Figure 1A). At a dose of 24 mg/kg, METH was effective in reducing the survival rate by 60% within 20 min; increasing the doses to 48 or 60 mg/kg further exacerbated the mortality rate to 90% or 100% by 20 min. In rats that died, there was a dose-dependent collapse of arterial pressure (Figure 1B) and heart rate (Figure 1C) that paralleled the time-course of mortality, with a progressive reduction in response latency. The LF component of the systolic blood pressure (SBP) spectrum underwent a transient increase that exhibited a dose-dependent reduction in peak power and response latency (Figure 1D), followed by the disappearance of this life-and-death signal that also paralleled temporally with mortality. However, survival rate (Figure 1A) or cardiovascular responses (Figure 1B–D) were not affected by intravenous administration of saline (vehicle control).

### Anoxia and cessation of tissue perfusion in RVLM

**Figure 2:**
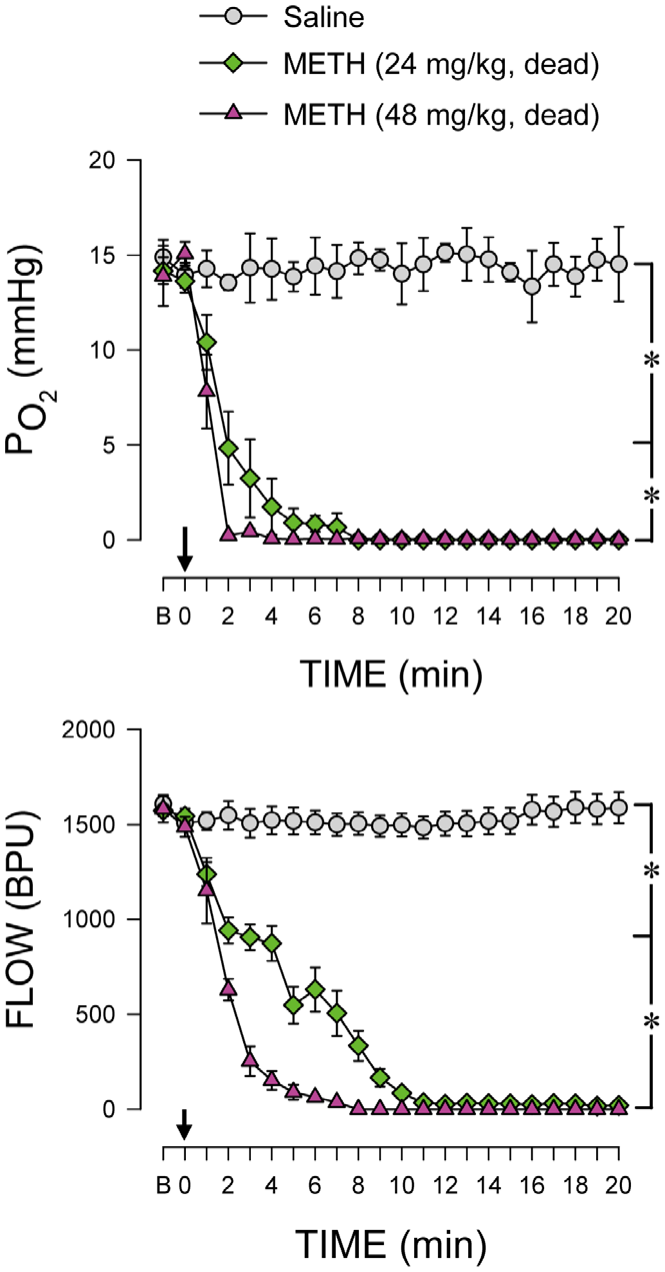
Methamphetamine dose-dependently and time-dependently elicited anoxia and cessation of microperfusion in rostral ventrolateral medulla. Temporal changes in tissue partial pressure of oxygen and blood flow detected in rostral ventrolateral medulla (RVLM) of animals that died of intravenous administration of METH (at arrow). Values are mean ± SEM, n = 5–9 animals per experimental group. *P<0.05 versus saline group at corresponding time-points in the post hoc Scheffé multiple-range test. B, baseline.

Our laboratory demonstrated previously [14] that anoxia and zero tissue perfusion in RVLM precede sustained cessation of LF power and cardiovascular collapse seen during experimental brain death. Figure 2 shows that in rats that succumbed to METH (24 or 48 mg/kg, i.v.), there was also an abrupt anoxia and cessation of local microvascular perfusion in RVLM. Of note was that those events exhibited a latency that was shorter than the cardiovascular responses induced by the same doses of METH. On the other hand, administration of saline induced insignificant changes in tissue oxygen level and local blood flow in RVLM. On the other hand, simultaneous measurement of in situ temperature revealed insignificant differences in RVLM tissue temperature between saline-control group (34.5±0.2°C), rats that survived after METH at 24 mg/kg (34.8±0.3°C) and rats that died of METH at 24 (33.8±0.5°C) or 48 mg/kg (33.7±0.3°C).

### RVLM as a site of action for intravenously administered METH

**Figure 3:**
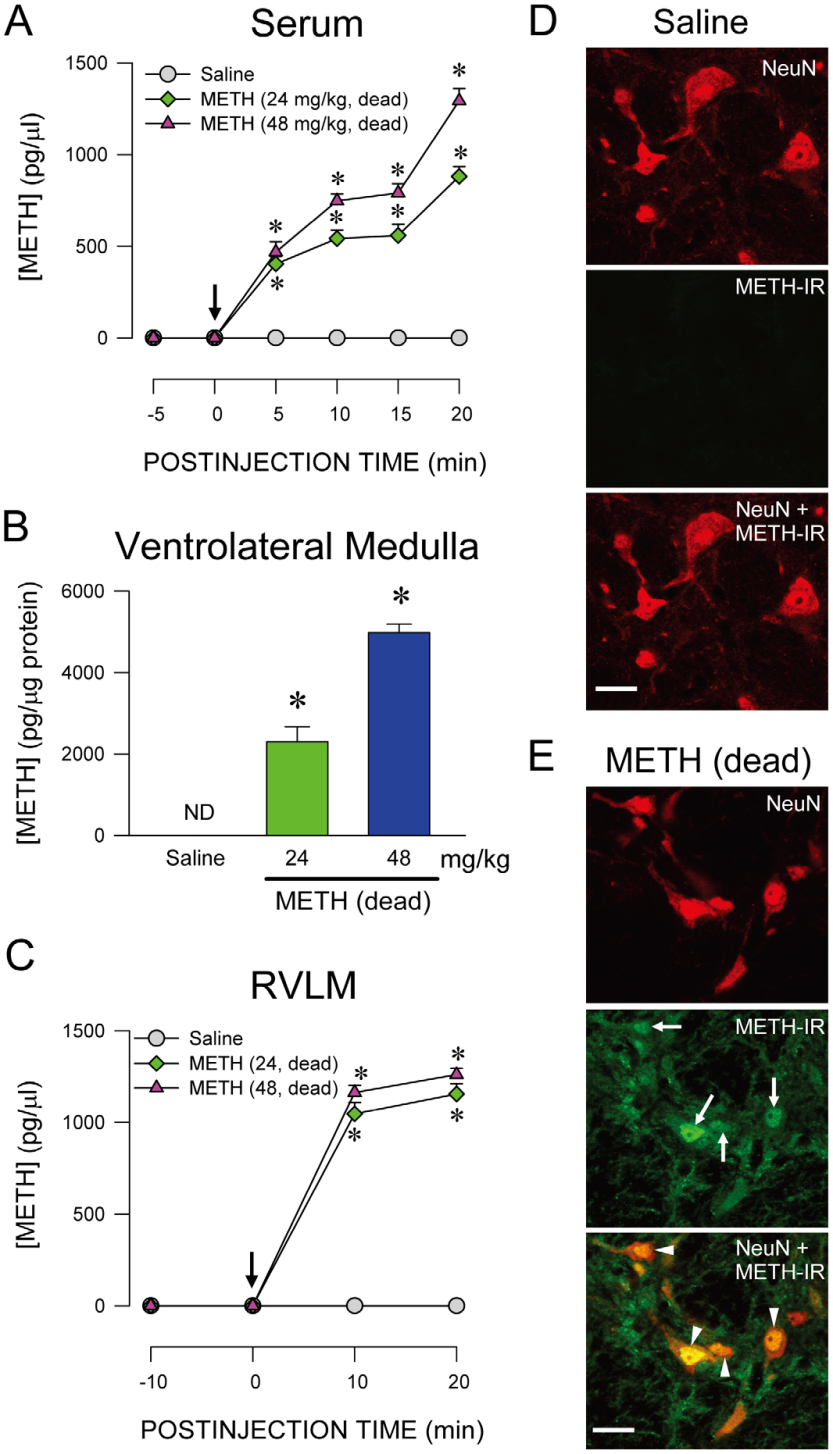
Presence of methamphetamine in RVLM neurons after intravenous administration. (A–C). Changes in concentration of METH in serum (A), ventrolateral medulla (B) or extracellular fluid collected from RVLM (C) of rats that died of intravenous administration of METH (at arrow). Values are mean ± SEM, n = 4–9 animals per experimental group. *P<0.05 versus saline group at corresponding time-points in the post hoc Scheffé multiple-range test. ND, not detected. (D–E). Representative laser scanning confocal microscopic images showing cells in RVLM that were immunoreactive to METH (METH-IR; green fluorescence) and additionally stained positively for a neuronal marker, neuron-specific nuclear protein (NeuN; red fluorescence) in rats that received saline (D) or died of METH (48 mg/kg, i.v.; E) group. These results are typical of 4 animals from each experimental group. Scale bar, 20 µm. White arrow, METH-IR cells in RVLM; white arrowhead, RVLM neurons which were double stained with METH-IR and NeuN.

A crucial prerequisite for RVLM to be a site of action for METH is for the psychostimulant to reach this neural substrate on systemic administration. In addition to a dose-dependent increase of METH in serum (Figure 3A), we detected the same trend in tissue samples (Figure 3B) and extracellular fluid (Figure 3C) collected from RVLM in rats that died of intravenous administration of METH (24 or 48 mg/kg). Double immunofluorescence staining coupled with laser scanning confocal microscopy further showed that in addition to a clearly defined nucleus and nucleolus in cells stained positively with the neuronal marker, neuron-specific nuclear protein (NeuN) (Figures 3D,E), METH-immunoreactivity was present in the cytoplasm and nucleus of RVLM neurons (Figure 3E) from animals that died of METH.

### Necrotic cell death in RVLM

**Figure 4:**
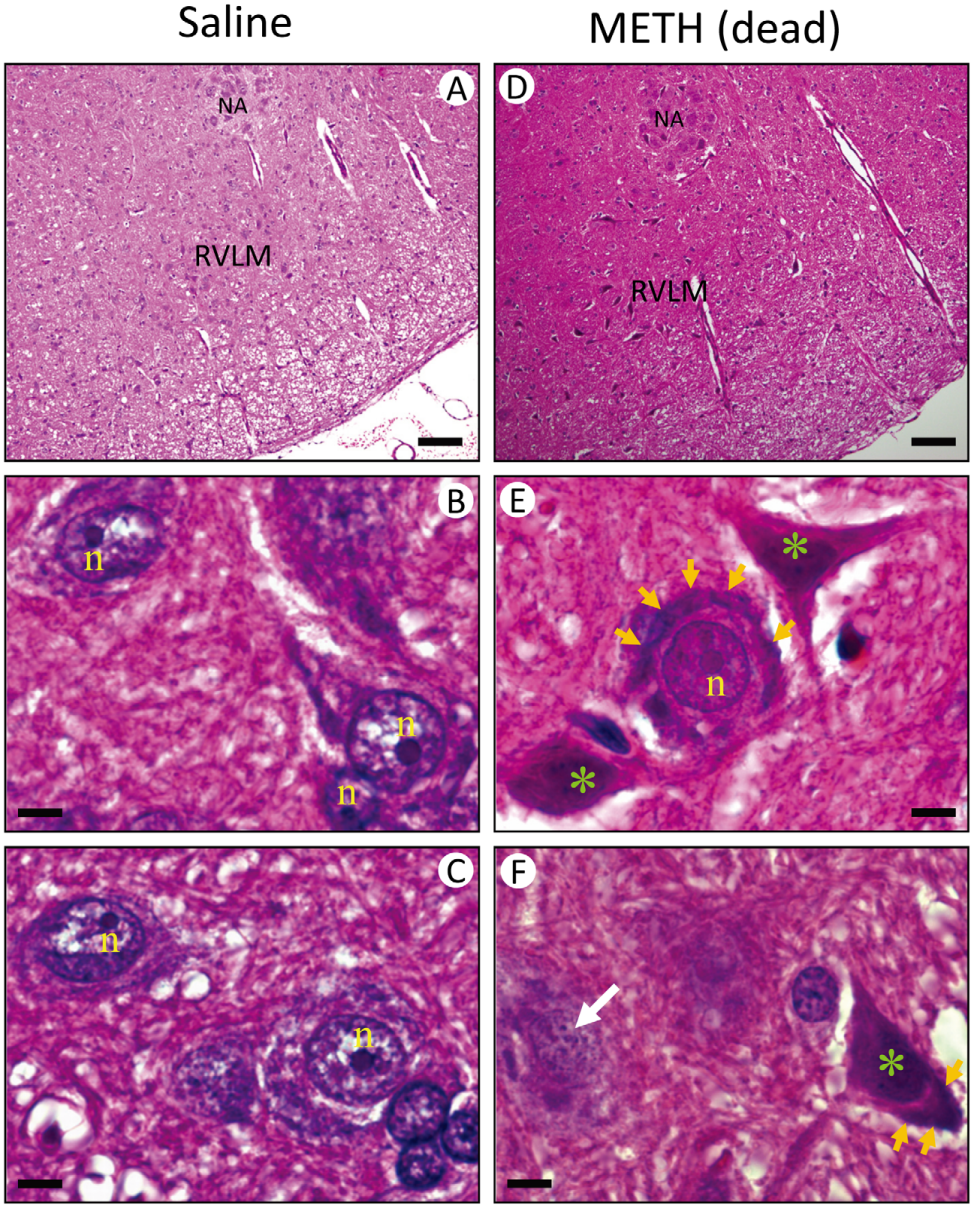
Methamphetamine induced necrotic cell death in RVLM. Representative photomicrographs of tissues stained by hematoxylin and eosin (H&E) showing nuclear pyknosis and chromatolysis in RVLM neurons of rats that received saline (A–C) or died of METH (48 mg/kg, i.v.) (D–F). These results are typical of 4–5 animals from each experimental group. Scale bar, 5 µm. NA, nucleus ambiguus; n, nucleus; yellow arrow, chromatolysis in RVLM neuron; white arrow, karyorrhexis in RVLM neurons; green symbol (*), karyolysis in RVLM neuron.

**Figure 5:**
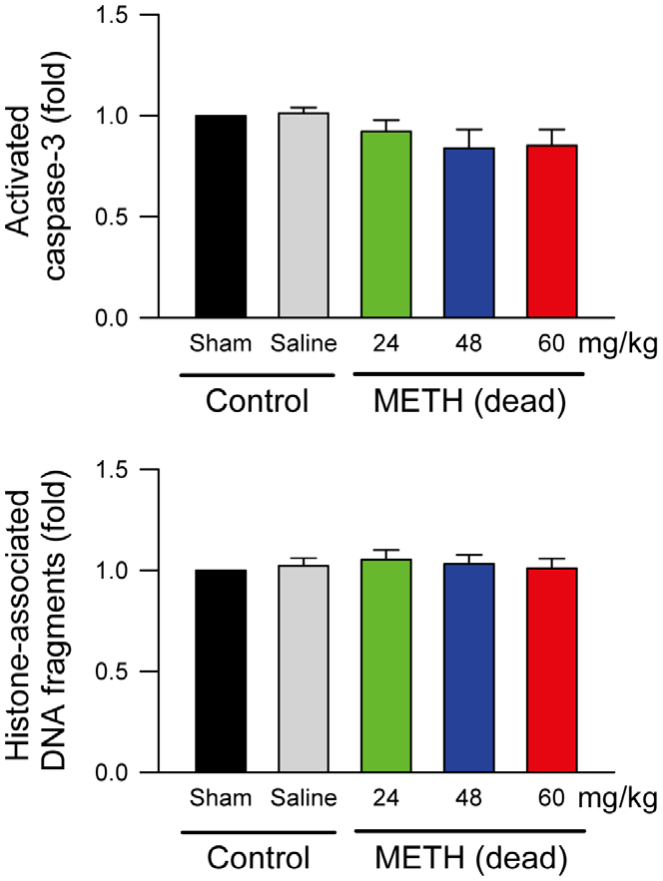
Methamphetamine did not induce apoptotic cell death in RVLM. Changes of activated caspase-3 or histone-associated DNA fragments in fold relative to sham-control group in samples collected from RVLM of rats that received saline or died of intravenous administration of METH. Values are mean ± SEM, n = 5–7 animals per experimental group. *P>*0.05 among all groups in one-way ANOVA.

Hematoxylin and eosin (H&E) staining revealed that present in RVLM of rats that died of METH (48 mg/kg, i.v.) were necrosis-appearing cells (Figure 4) characterized by shrinkage of cytoplasm, chromatolysis (disruption of Nissl bodies), karyolysis (nuclear fading) and karyorrhexis (nuclear fragmentation). On the other hand, 81.9±1.1% of cells in RVLM of saline-control rats exhibited clearly hematoxylin-labeled nucleolus, intact nuclear membrane, nucleus and cytoplasm were observed (Figure 4), as opposed to 13.8±1.9% in the METH (48 mg/kg, i.v.) group. Likewise, the ratio of RVLM cells that manifested chromatolysis (23.7±1.2% versus 3.7±0.7%), karyolysis (40.6±2.5% versus 7.6±1.1%) and karyorrehexis (21.9±1.4% versus 6.8±0.5%) in the METH group was higher than those in the saline-control group. In contrast, activated caspase-3 and histone-associated DNA fragments, two hallmark experimental indices for apoptosis, were absent from RVLM of rats that received saline or died of METH (Figure 5).

### Bioenergetic failure that leads to necrotic cell death because of mitochondrial dysfunction in RVLM

**Figure 6:**
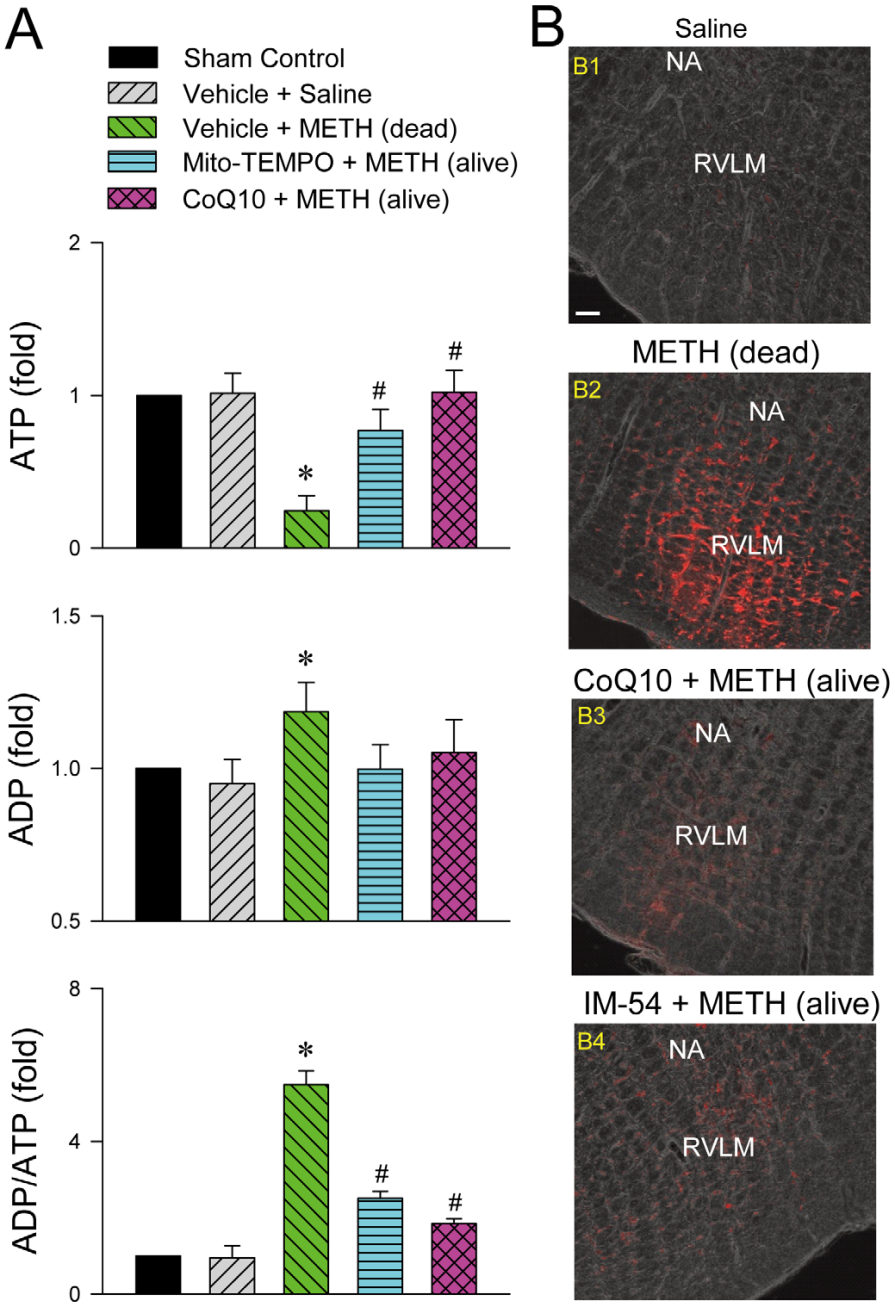
Methamphetamine induced bioenergetics failure in RVLM. (A). Fold changes relative to sham-control group in the level of ATP, ADP or ADP/ATP ratio in samples collected from RVLM of rats that died of METH (24 mg/kg, i.v.) or survived with pretreatment by microinjection of Mito-TEMPO (500 pmol) or coenzyme Q10 (CoQ10; 7 nmol) into RVLM prior to METH administration. Values are mean ± SEM, n = 3–5 animals per experimental group. *P<0.05 versus Vehicle+Saline group, and^ #^ *P*<0.05 versus Vehicle+METH group in the post hoc Scheffé multiple-range test. (B). Representative laser scanning confocal microscopic images superimposed on phase contrast images showing neurons in RVLM that were immunoreactive to propidium iodide in rats that received saline (B1), died of METH (24 mg/kg, i.v.; B2), or survived with pretreatment by microinjection of CoQ10 (7 nmol; B3) or IM-54 (3 pmol; B4) into bilateral RVLM prior to METH administration. These results are typical of 4 animals from each experimental group. Scale bar, 100 µm. NA, nucleus ambiguus.

**Figure 7:**
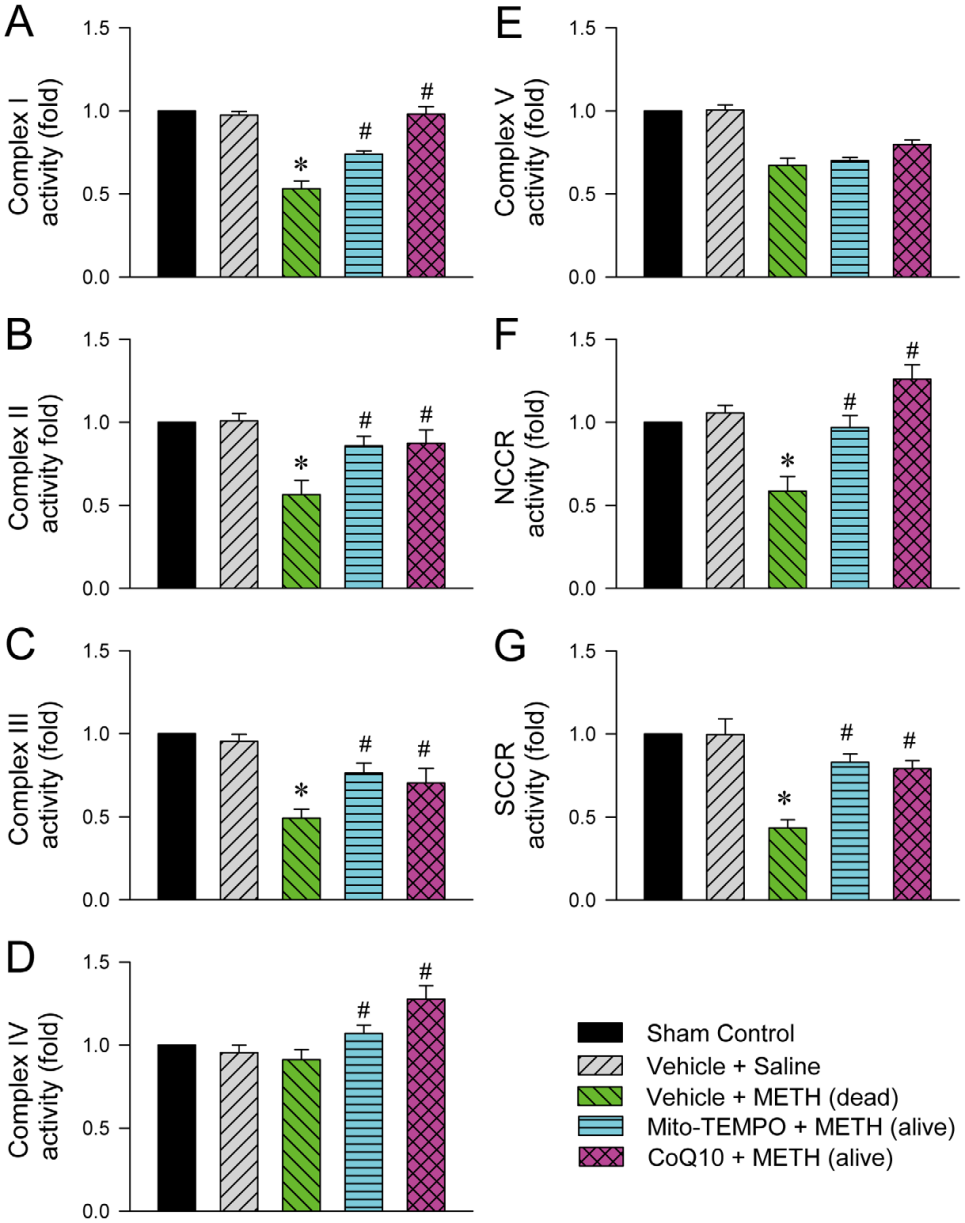
Methamphetamine induced mitochondrial dysfunction in RVLM. Fold changes relative to sham-control group in the activity of Complex I (A), II (B), III (C), IV (D) or V (E) and electron transfer capacity between Complexes I and III (NCCR; F)or II and III (SCCR; G) in the mitochondrial respiratory chain in samples collected from RVLM of rats died of METH (24 mg/kg, i.v.) or survived with pretreatment by microinjection of Mito-TEMPO (500 pmol) or coenzyme Q10 (CoQ10; 7 nmol) into bilateral RVLM prior to METH administration. Values are mean ± SEM, n = 4–5 animals per experimental group. *P<0.05 versus Vehicle+Saline group, and ^#^ *P*<0.05 versus Vehicle+METH group in the post hoc Scheffé multiple-range test.

Because ATP is the primary source of metabolic energy in neurons, neurons tend to undergo necrosis in response to anoxic stress [17]. We found that animals died of METH (24 mg/kg, i.v.) exhibited a drastic reduction in ATP production and an increase in ADP level or ADP/ATP ratio in RVLM (Figure 6A), alongside elevated PI uptake indicative of necrotic cell death (Figure 6B). In addition to maintaining survival, all those four responses were reversed by microinjection bilaterally of Mito-TEMPO (500 pmol) or CoQ10 (7 nmol) into RVLM (Figures 6A,B); and IM-54 (3 pmol) blunted the elevated PI level (Figure 6B). We further observed a depression of the activities of Complexes I, II, III and V (Figures 6A–C,E), NADH cytochrome *c* reductase (NCCR) or succinate cytochrome *c* reductase (SCCR) (Figures 7F,G), but not Complex IV (Figure 6D), in the mitochondrial respiratory chain in RVLM of rats that succumbed to METH (24 mg/kg, i.v.). Again, those depressive actions were antagonized by CoQ10 (7 nmol) or Mito-TEMPO (500 pmol) (Figures 7A–C,E–G).

### Increase of mitochondrial superoxide anion in RVLM

**Figure 8:**
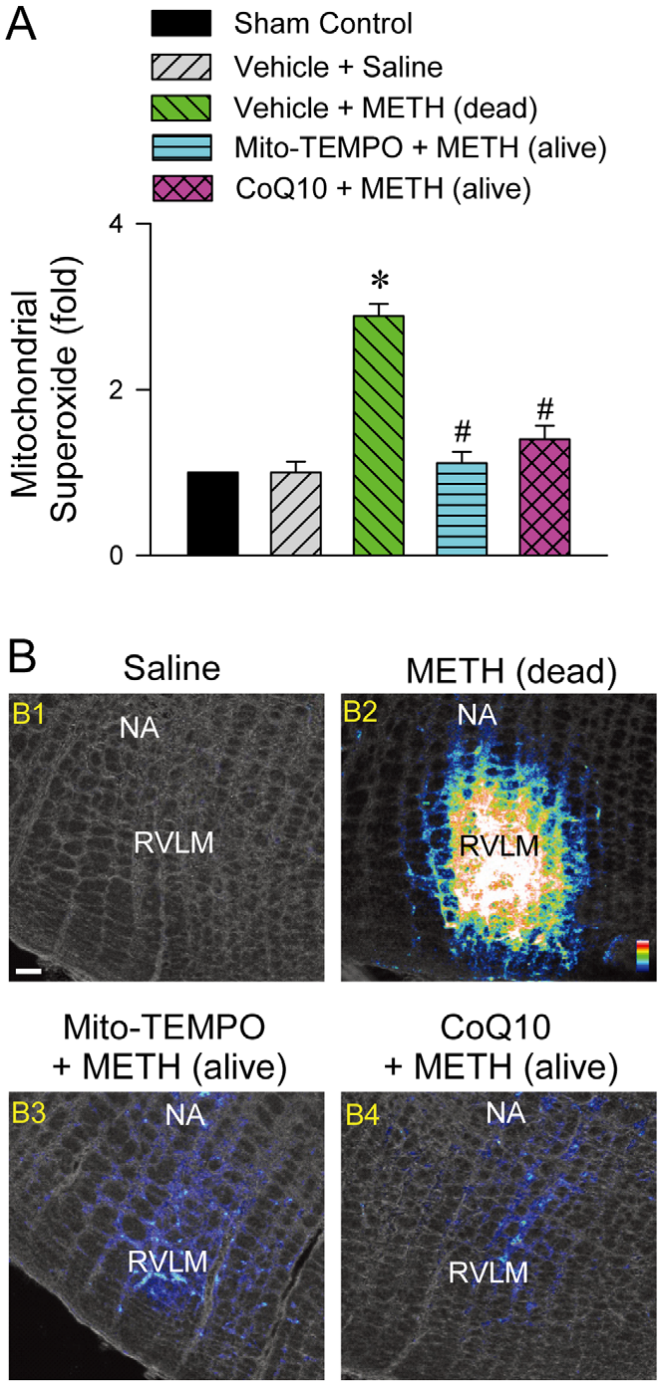
Methamphetamine increased production of mitochondrial superoxide anions in RVLM. (A). Fold changes relative to sham-control group in mitochondrial superoxide anion in sample collected from RVLM of rats died of METH (24 mg/kg, i.v.) or survived with pretreatment by microinjection of Mito-TEMPO (500 pmol) or coenzyme Q10 (CoQ10; 7 nmol) into RVLM prior to METH administration. Values are mean ± SEM, n = 3–6 animals per experimental group. *P<0.05 versus Vehicle+Saline group, and ^#^ *P*<0.05 versus Vehicle+METH group in the post hoc Scheffé multiple-range test. (B). Representative laser scanning confocal microscopic images superimposed on phase contrast images showing neurons in RVLM that were immunoreactive to MitoSOX in rats that received saline (B1), died of METH (24 mg/kg, i.v.; B2), or survived with pretreatment by microinjection of Mito-TEMPO (500 pmol; B3) or CoQ10 (7 nmol; B4) into bilateral RVLM prior to METH administration. These results are typical of 4 animals from each experimental group. Scale bar, 100 µm. Pseudocolor scale bar, white color represented highest and blue color represented lightest fluorescence intensity. NA, nucleus ambiguus.

In rats that died of METH (24 mg/kg, i.v.), there was also an increase of mitochondria-derived superoxide anion level in RVLM as demonstrated by enzyme-linked immunosorbent assay (ELISA) (Figure 8A) or represented by the intensity of fluorescence from a mitochondrial superoxide indicator (Tyagi *et al.*, 2010), MitoSOX (Figure 8B). Microinjection bilaterally of CoQ10 (7 nmol) or Mito-TEMPO (500 pmol) again significantly blunted those responses (Figures 8A,B). On the other hand, negligible superoxide anion was observed in RVLM (Figures 8A,B) of sham-controls or saline-treated rats.

### Mitochondrial dysfunction, bioenergetic failure, oxidative stress and necrotic cell death in RVLM underlie METH-induced cardiovascular collapse and mortality

**Figure 9:**
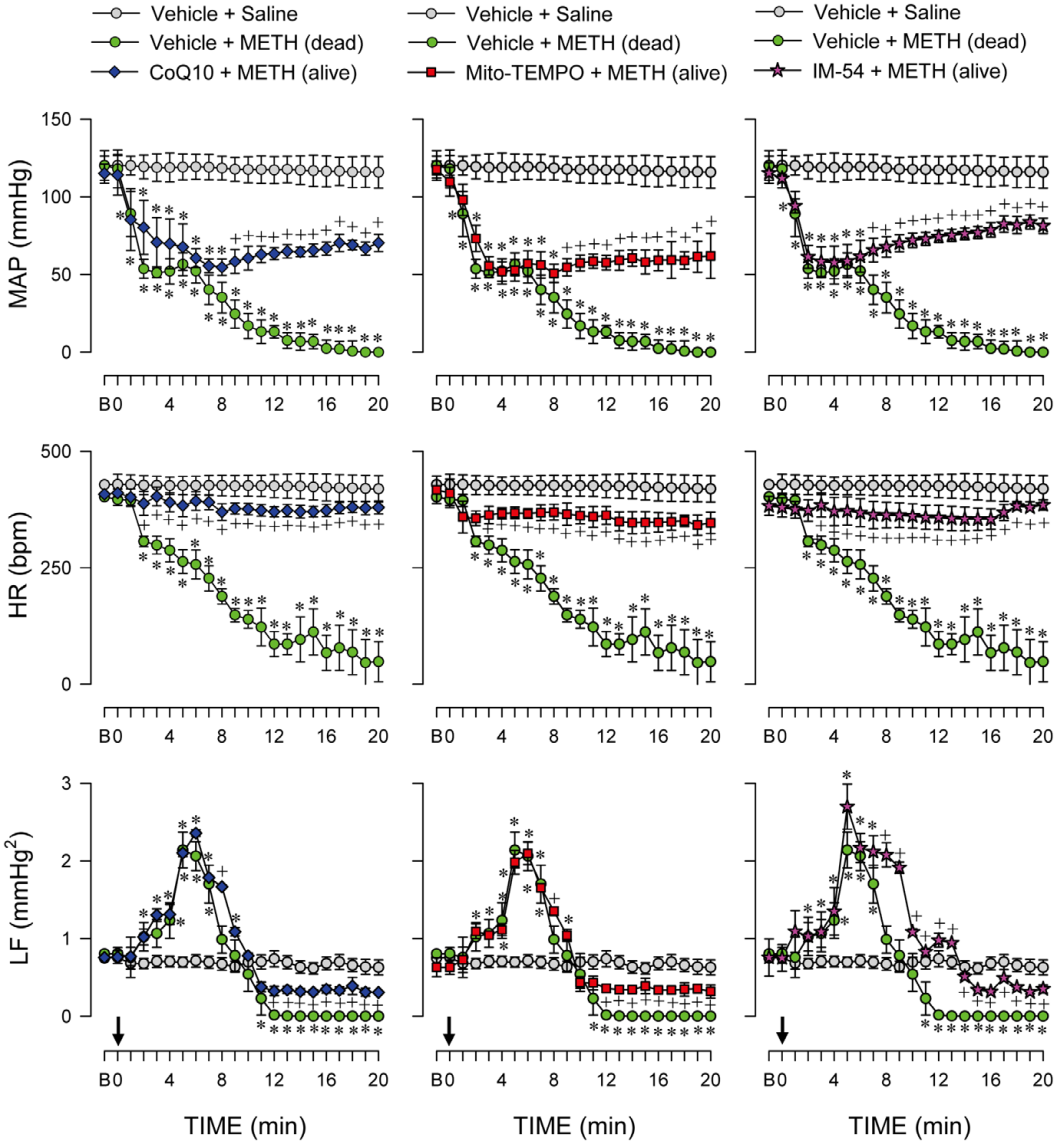
Oxidative stress in RVLM underlied methamphetamine-elicited cardiovascular collapse. Temporal changes in MAP, HR or power density of the LF component of systolic blood pressure signals in rats that received saline, died of METH (24 mg/kg, i.v.) or survived with pretreatment by microinjection of CoQ10 (7 nmol), Mito-TEMPO (500 pmol) or IM-54 (3 pmol) into bilateral RVLM prior to METH administration. Values are mean ± SEM, n = 6–37 animals per experimental group. *P<0.05 versus Vehicle+Saline group, and ^+^ *P*<0.05 versus Vehicle+METH group in the post hoc Scheffé multiple-range test.

Microinjection of CoQ10, Mito-TEMPO or IM-54 bilaterally into RVLM, at a dose that antagonized mitochondrial dysfunction, bioenergetic failure, oxidative stress or necrotic cell death in RVLM (Figure 9), significantly sustained survival (vehicle+METH, 40%; CoQ10+METH, 78%; Mito-TEMPO+METH, 70%; IM-54+METH, 85%) by antagonizing arterial pressure collapse, asystole and disappearance of power density of the LF spectral component induced by intravenous administration of a lethal dose of METH (24 mg/kg).

## Discussion

**Figure 10:**
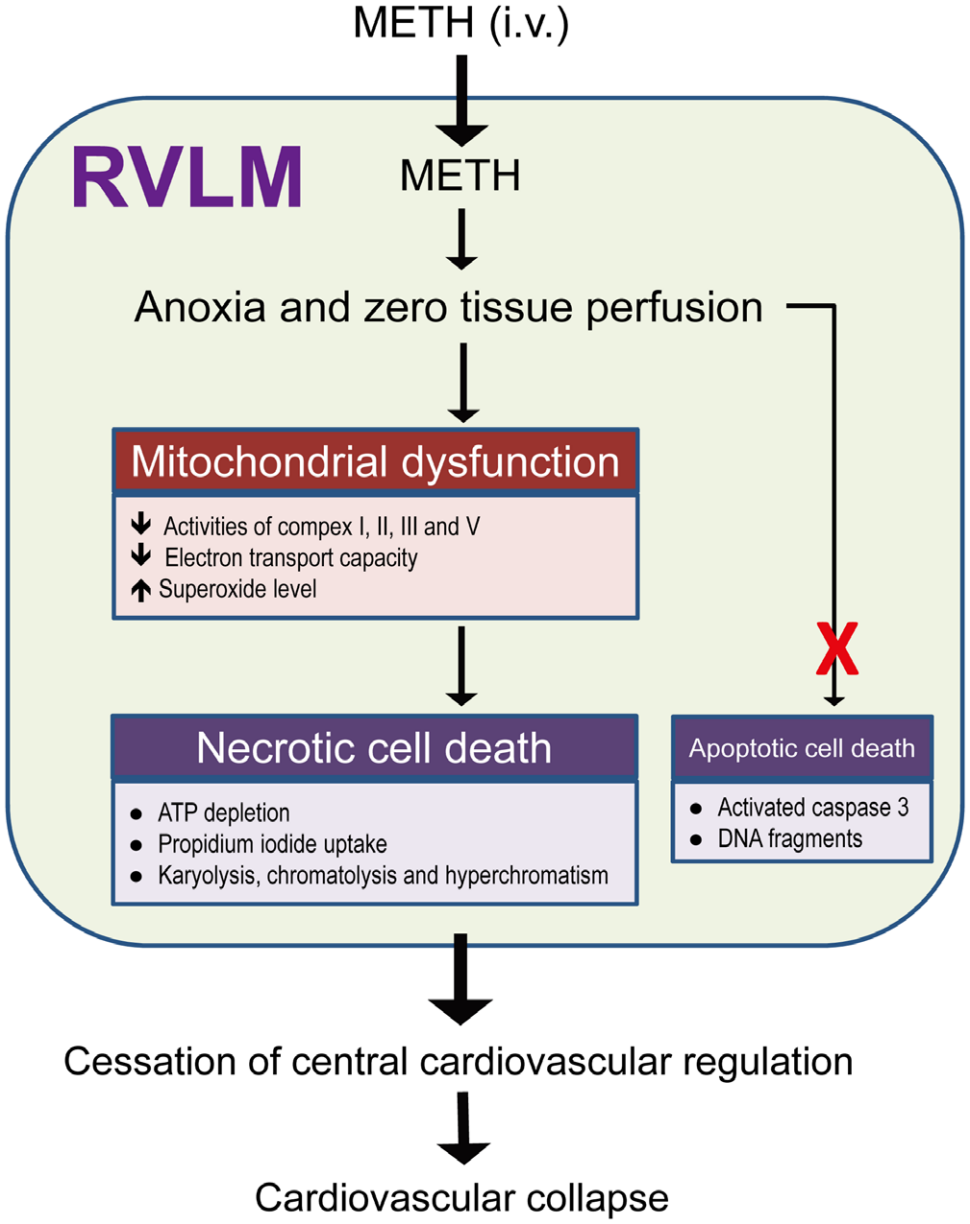
Proposed mechanisms in RVLM that underlies METH-induced cardiovascular collapse. Intravenous administration of METH rapidly reaches RVLM to induce anoxia and cessation of tissue perfusion, followed by bioenergetics failure and oxidative stress because of mitochondrial dysfunction that lead to necrotic cell death. The loss of functionality in RVLM results in cessation of central cardiovascular regulation and the eventual cardiovascular collapse.

A majority of the investigations on METH focuses on the behavioral alterations, physical dependence or psychopathology of withdrawal syndrome and its management that are associated with this psychostimulant. Much less studies are devoted to the mechanisms of METH intoxication despite that it is a common cause of death within the abuse population [4], [5]. Based on a fatal METH intoxication model, the present study provided the first demonstration that acute cessation of brain stem cardiovascular regulation that resembles that in brain death because of necrotic cell death in RVLM underpins METH-induced cardiovascular collapse. We further demonstrated that the repertoire of interposing cellular events triggered by METH in RVLM includes sustained anoxia and zero tissue perfusion that leads to bioenergetics failure and oxidative stress because of mitochondrial dysfunction (Figure 10).

The experimental design of the present study addresses specifically the mechanisms of METH-induced cardiovascular collapse rather than to model consequences of human METH abuse. As such, we chose intravenous administration of METH in our study because this is a common route [18] that is employed by abusers to rapidly raise the blood concentration of METH to euphoric levels. At the same time, we chose to employ high doses of METH that would result in cardiovascular collapse and fatality to mimic observations from clinical conditions [19], [20]. Compared to its primary metabolite amphetamine, METH is more lipid-soluble, with enhanced transport across the blood-brain barrier and stability against enzymatic degradation by monoamine oxidase [21]. Indeed, our pharmacokinetic data indicated that 10 min after intravenous administration, the concentration of METH in the extracellular microenvironment of RVLM was already 10 times higher than that in serum, approaching a plateau within 20 min. With the high tissue concentration detected in ventrolateral medulla and the demonstrated presence of METH in RVLM neurons, the present study established RVLM as a relatively unknown though vital brain targets for this psychostimulant.

The present results were highly reminiscent of those in a recent study from our laboratory [14] that showed that a crucial determinant for successful revival of an arrested heart is that spontaneous circulation must resume before brain death commences, and that maintained functional integrity of RVLM holds the key to this vital time-window. Our results showed that a time-window represented experimentally by the power density of the LF component of the SBP spectrum also exists between the administration of METH and death, and was inversely related to the doses used. We further demonstrated that sustained anoxia, which took place as early as 2 min after intravenous administration of METH at 48 mg/kg, followed 4–6 min later by cessation of tissue perfusion, accounts for the loss of functionality of RVLM. Of note is that this time-window parallels the time taken for extracellular concentration of METH in RVLM to reach plateau. Given that RVLM is long known to be responsible for the maintenance of sympathetic vasomotor tone and stable blood pressure [13], and is the origin of a life-and-death signal [12] that disappears in patients confirmed of brain death [11], the selective vulnerability of this neural substrate to METH-induced toxicity is to be anticipated.

Neuronal functions are critically dependent on a continuous supply of oxygen because their primary source of metabolic energy is oxidative phosphorylation in the mitochondrion. The mitochondrial respiratory chain located in the inner membrane of mitochondria comprises of Complexes I, II, III, IV and V (ATP synthase). Orderly transfer of electrons through this chain produces energy in the form of ATP. A disruption of electron transfer not only decreases ATP production but also leaks electrons from the inner membrane to the matrix that results in the formation of reactive oxygen species (ROS) primarily in the form of superoxide anions. The mitochondrion is also the primary site of oxygen consumption in the cell, and as such is highly susceptibility to anoxia. The drastic reduction in ATP production and increase in ADP level or ADP/ATP ratio, alongside significant depression of the activity of most of the mitochondrial respiratory chain Complexes, reduction of the capacity of electron transport between Complexes I to III (NCCR) and II to III (SCCR), and augmentation of mitochondria-derived superoxide anion in RVLM of rats that died of METH strongly support the notion that bioenergetic failure and oxidative stress triggered by anoxia and cessation of tissue perfusion precipitate the eventual cessation of neuronal functions in RVLM. We noted that METH induces mitochondrial damage via production of ROS in rat hippocampal neural progenitor cells [22] and human dopaminergic neuroblastoma SH-SY5Y cells [23]. Aberrant mitochondrial biogenesis and enhanced oxidative stress also play a crucial role in METH-induced neurotoxic effects in SH-SY5Y cell line [24]. Depressive effects of METH on the activity of mitochondrial respiratory chain complexes have also been reported [25]. In spontaneously hypertensive rats, the increased superoxide anions in RVLM can also take origin from NADPH oxidase [26]. Whether this source of ROS is involved in METH-induced oxidative stress in RVLM requires further elucidation.

Postmortem studies showed that cerebral edema [27] or brain stem hematoma [15] is a common clinical phenotype in patients died of METH intoxication. The present study demonstrated that damage to RVLM in the form of necrotic cell death is a contributing factor to METH-induced mortality. A critical determinant of the eventual cell death fate resides in intracellular ATP concentration. Whereas ATP is required for the development of apoptosis, ATP depletion is associated with necrosis [28]. Thus, the significant depletion of intracellular ATP content induced by lethal doses of METH, together with the characteristic morphological features under H&E stain and the uptake of PI, are indicative of damage of cell membrane integrity, leading to necrotic cell death in RVLM. The absence of activated caspase-3 and histone-associated DNA fragments, two hallmark experimental indices for apoptosis, in RVLM of rats that died of METH further supports the validity of this notion. We previously found that lethal dose of organophosphate pesticide mevinphos induced anoxia-induced bioenergetics failure and subsequent necrotic cell death in RVLM, resulting in cardiovascular collapse prior to cessation of life. On the other hand, sub-lethal dose of mevinphos elicited hypoxia-dependent and mitochondria-dependent apoptotic cell death in RVLM that result in dysfunction of brain stem cardiovascular regulation and hypotension [29]–[32]. It follows that, subject to confirmation, sub-lethal doses of METH may only induce hypoxia in RVLM, leading to apoptotic cell death that is accompanied by brain stem cardiovascular dysfunction without imminent death.

In addition to mitochondria, the endoplasmic reticulum is reported recently to be another target of ROS [33]. Whereas the contribution of endoplasmic reticulum to METH-induced necrotic cell death in RVLM cannot be ruled out, we noted that ROS-mediated endoplasmic reticulum stress is mainly responsible for apoptosis induced by proapoptotic inducer in cancer cells [33], [34]. Because of the similarities in cellular programming for apoptosis and autophagy [35], [36], the lack of participation of apoptotic cell death in RVLM during METH-induced cardiovascular collapse observed in the present study deemed the involvement of autophagy in this process minimal.

The effectiveness of CoQ10, Mito-TEMPO or IM-54 to sustain survival, antagonize arterial pressure collapse, asystole and disappearance of power density of the LF spectral component, and blunt mitochondrial dysfunction, bioenergetics failure, oxidative stress or necrotic cell death in RVLM induced by lethal doses of METH provided the most crucial documentation that these events are causally related. CoQ10 is an essential component of the mitochondrial electron transport chain [37], and the concentration of CoQ10 in the mitochondrial membrane is related to the rate of electron transfer and respiratory function [38]. By enhancing quantitatively the availability of freely mobile electron carriers and qualitatively the efficacy of electron transfer across the mitochondrial respiratory chain [39], CoQ10 may also avail more oxygen to the neurons for oxidative phosphorylation. Mito-TEMPO is a recently developed mitochondria-targeted antioxidant and superoxide dismutase mimetic [40] that prevents loss of mitochondrial membrane potential or increase in mitochondrial permeability transition pore opening and necrosis in epithelial cells that are subject to ATP depletion [41]. Mito-TEMPO also decreases mitochondrial superoxide level, prevents mitochondrial oxidative damage or mitochondrial dysfunction and attenuates hypertension elicited in mice by angiotensin II or DOCA salt [40]. IM-54 is a recently developed tool for investigating the molecular mechanism of oxidative stress-induced necrosis. It inhibits necrotic cell death in hydrogen peroxide-treated HL60 cells or in anticancer agent chrysophanol-treated J5 human liver cancer cells [42], but not in etoposide-induced apoptotic cell death [43].

We are aware that heart failure is another common phenomenon observed in patients died of METH, and METH may act directly on the heart on intravenous administration [6], [8], [9]. As such, the possibility exists for depression of cardiac functions, rather than failure of brain stem cardiovascular regulation, to be primarily responsible for our observed METH-induced cardiovascular collapse. However, our results deemed this possibility minimal. Pretreatments with CoQ10, Mito-TEMPO or IM-54, at a dose that antagonized mitochondrial dysfunction (Figure 7), bioenergetic failure (Figure 6), oxidative stress (Figure 8) or necrotic cell death (Figure 6) in RVLM, significantly antagonized the cessation in arterial pressure or heart rate and disappearance of the LF spectral component (Figure 9) induced by intravenous administration of a lethal dose of METH, along with sustained survival.

Subcutaneous administration of METH (9 mg/kg) has been reported to elicit hyperthermia and structural or functional abnormalities in rat nucleus accumbens [44], cortex, hippocampus, thalamus and hypothalamus [45]. In particular, acute brain edema induced by the inflow of simple ions promoted by a breakdown of blood-brain barrier presents serious consequences [46]. Since animals that died of METH did not manifest significant changes of tissue temperature in RVLM, we reason that the neurotoxicity we observed in this neural substrate may not be the result of local hyperthermia. On the other hand, it is plausible that the cellular consequences induced by METH in the forebrain structures may also take place in RVLM. Further experiments are required to address this issue.

In conclusion, the present study revealed that lethal doses of METH elicit cardiovascular collapse by inducing an acute loss of functionality in RVLM via necrotic cell death, which leads to the cessation of brain stem cardiovascular regulation that resembles that in brain death. We further showed that the underlying cellular events include mitochondrial dysfunction that leads to depletion of ATP production (bioenergetic failure) and upregulation of mitochondrial superoxide anion (oxidative stress) because of sustained anoxia and zero tissue perfusion in RVLM. This information should offer novel leads for devising clinical management or developing therapeutic strategies against the increasing number of sudden death from METH abusers. Since the mechanisms that we identified for METH-induced cardiovascular collapse are also shared by other pathologies, including brain death and resuscitation of an arrested heart, our observations may have even broader clinical ramifications.

## Materials and Methods

### Ethics statement

All experimental procedures carried out in this study were approved by the Institutional Animal Care and Use Committee of the Kaohsiung Chang Gung Memorial Hospital (CGMH891071), and were in compliance with the guidelines for animal care and use set forth by that committee. All efforts were made to reduce the number of animals used and to minimize animal suffering during the experiment.

### Animals

Specific pathogen-free adult, male, Sprague-Dawley rats (260 to 325 g, n = 200) purchased from the Experimental Animal Center of the National Science Council and BioLASCO, Taiwan, Republic of China, were used. They were housed in an Association for Assessment and Accreditation of Laboratory Animal Care (AAALAC) International-accredited animal facility under temperature control (24–25°C) and 12-h light-dark cycle. Standard laboratory rat chow and tap water were available ad libitum.

### General preparation

Under an induction dose of pentobarbital sodium (50 mg/kg, i.p.), rats received preparatory surgery that included tracheal intubation and cannulation of the femoral artery and vein. Animals received thereafter an intravenous infusion of propofol (20–25 mg/kg/h; Zeneca, Macclesfield, England), which provided satisfactory maintenance of anesthesia while preserving the capacity of central cardiovascular regulation [47]. During the recording sessions, animals were allowed to breathe spontaneously with room air, and body temperature was maintained at 37°C by a heating pad.

### Recording of cardiovascular parameters

Arterial pressure (AP) signals recorded from the femoral artery were processed by an arterial blood pressure analyzer (APR31a; Notocord, Croissy-Sur-Seine, France) and the SBP signals were subject simultaneously to online and real-time spectral analysis (SPA10a; Notocord). We were particularly interested in the LF (0.25–0.8 Hz) component because it takes origin from RVLM [12], and its power density mirrors the prevalence of baroreflex-mediated sympathetic neurogenic vasomotor discharges that emanate from RVLM [48] and is therefore a reasonable index for brain stem cardiovascular regulation. With particular reference to the present study, the increase or decrease in LF power was also used to reflect lower or higher probability of manifesting brain death [11], [49]–[51]. Heart rate (HR) was derived instantaneously from AP signals.

### Intravenous administration of METH

METH was purchased from the Food and Drug Administration, Department of Health, Executive Yuan, Taipei, Taiwan. It was administered intravenously, with saline serving as the vehicle control. Temporal changes in pulsatile AP, mean AP (MAP), HR and power density of the LF component were routinely followed for 20 min in an on-line and real-time manner [14], [16], [52], or until the animal succumbed to METH. The survival rate within 240 min was also recorded.

### Measurement of tissue oxygen tension, microvascular perfusion and temperature in rostral ventrolateral medulla

Simultaneous and continuous measurement of tissue oxygen tension, blood flow and temperature (Oxford Optronix, Oxford, England) was carried out by a combined oxygen/temperature/blood flow probe stereotaxically positioned into the RVLM [14], [52]. The coordinates used were: 4.5 to 5 mm posterior to lambda, 1.8 to 2.1 mm lateral to midline and 8.1 to 8.4 mm below dorsal surface of cerebellum. Instantaneous changes in local oxygen tension, compensated for fluctuations in tissue temperature, were processed by an OxyLite monitor (Oxford Optronix). Real-time microvascular red blood cell perfusion in tissue was processed by an OxyFlo monitor (Oxford Optronix). Laser Doppler signals from the tissue were recorded in blood perfusion units (BPU), which is a relative unit defined against a controlled motility standard.

### Collection of tissues from RVLM

We routinely collected tissue samples [52]–[54] immediately after rats died of METH or 20 min after intravenous administration of saline in rats killed with an overdose of pentobarbital sodium. Tissue samples from both sides of the ventrolateral medulla, at the level of RVLM (0.5–1.5 mm rostral to the obex), were collected by micropunches made with a 1-mm (i.d.) stainless-steel bore to cover the anatomical boundaries of RVLM, with a diameter of 625–650 µm [52]. In some experiments, medullary tissues collected from anesthetized animals but without receiving experimental manipulations served as the sham controls.

### Determination of METH in serum, ventrolateral medulla or extracellular fluid from RVLM

The level of METH in serum, ventrolateral medulla or perfusate collected from RVLM was determined by a commercial METH indirect ELISA kit (Calbiotech, Spring Valley, CA, USA), in conjunction with a spectrophotometer (Multiskan Spectrum, Thermo Fisher Scientific Waltham, MA, USA) [55], [56]. Blood (100 µl) drawn from the femoral vein was centrifuged to obtain serum samples, which were frozen at −80°C until analysis. The perfusate from the extracellular space of RVLM was continuously collected at 10-min interval via a microdialysis probe (CMA/Microdialysis, Stockholm, Sweden) stereotaxically positioned into RVLM and perfused with artificial cerebrospinal fluid (aCSF) at a rate of 1 µl/min.

### Immunofluorescence staining and confocal microscopy

We employed double immunofluorescence staining coupled with laser scanning confocal microscopy [14], [57], [58] to detect localization of METH in RVLM neurons using a rabbit polyclonal antiserum against METH (Abcam, Cambridge, MA, USA), and a mouse monoclonal antiserum against a specific neuron marker, NeuN (Millipore, Billerica, MA, USA). The secondary antisera (Molecular Probes, Eugene, OR, USA) used included a goat anti-rabbit IgG conjugated with Alexa Fluor 488 for METH, and a goat anti-mouse IgG conjugated with Alexa Fluor 568 for NeuN. Tissues similarly processed but omitting primary antiserum against METH served as our negative controls. Immunoreactivity was viewed under a Fluorview FV10i laser scanning confocal microscope (Olympus, Tokyo, Japan).

### H&E staining

Brain stem fixed in 10% buffered formalin for 48 h were embedded in paraffin wax and processed for H&E staining [59], [60]. Four µm-thick tissue sections were dewaxed in xylene, hydrated in decreasing percentages of alcohol and stained with hematoxylin (Merck Millipore, Darmstadt, Germany). This was followed by dehydration in increasing percentages of alcohol until 70%, staining with 0.25% eosin (Merck Millipore) in alcohol, differentiation in 90% alcohol, clearing in xylene and finally coverslipped in mounting medium (Merck Millipore). The stained sections were observed and photographed under an optical microscope (Olympus).

### Propidium iodide (PI) staining

Uptake of PI by RVLM neurons as a marker for necrotic cell death [61], [62] was determined by the exhibition of red fluorescence under laser scanning confocal microscopy (Olympus). PI (5 pmol; Sigma-Aldrich, Saint Louis, MO, USA) was microinjected bilaterally into RVLM 30 min before METH or vehicle administration.

### Experimental indices for apoptosis

To quantify apoptosis, a cell death ELISA kit (Roche Applied Science, Indianapolis, IN, USA) that detects apoptotic but not necrotic cell death was used to measure the level of histone-associated DNA fragments in the cytoplasm [57], in conjunction with an ELISA microtiter plate reader (Anthros Labtec, Salzburg, Austria). To quantify activated caspase-3, a caspase-3 fluorescence assay kit (Cayman, Ann Arbor, MI, USA) coupled with a spectrofluorometer (Fluoroskan Ascent FL, Thermo Fisher Scientific Inc.) was used.

### Microinjection of test agents to RVLM

Microinjection bilaterally of test agents into RVLM, each at a volume of 50 nl, was carried out stereotaxically and sequentially via a glass micropipette connected to a 0.5-µl microsyringe (Hamilton, Reno, NV, USA) [16], [52], [58]. Test agents used included a mobile electron carrier in the mitochondrial respiratory chain [37], coenzyme Q10 (CoQ10); gift kindly from PharmaEssentia, Taipei, Taiwan); a mitochondria-targeted antioxidant and superoxide anion scavenger [40], [41], [(2-(2,2,6,6-tetramethylpiperidin-1-oxyl-4-ylamino)-2- oxoethyl)triphenylphosphonium chloride] (Mito-TEMPO; Enzo Life Sciences, Plymouth Meeting, PA, USA); an oxidative stress-induced necrotic cell death inhibitor [43], [63], 2-[1H-indol-3-yl]-3-pentylamino-maleimide (IM-54; Santa Cruz Biotechnology, Santa Cruz, CA, USA); a highly selective fluorogenic indicator for mitochondrial superoxide [64], MitoSOX red mitochondrial superoxide indicator (MitoSOX; Molecular probes) or PI (Sigma-Aldrich), with distilled water (solvent for CoQ10 and Mito-TEMPO) or 0.2% DMSO (solvent for IM-54 and MitoSOX) serving as the vehicle control. To avoid the confounding effects of drug interactions, each animal received only one freshly prepared pharmacological pretreatment.

### Measurement of ATP or ADP concentration

An ApoSENSOR ADP/ATP ratio assay (BioVision, Mountain View, CA, USA) was used to determine ATP or ADP concentration and ADP/ATP ratio [65]. Light emitted from a luciferase mediated reaction and measured by a luminometer (Berthold Detection Systems GmbH, Pforzheim, Germany) was used to calculate the measured values.

### Assay for mitochondrial electron transport chain complex enzyme activity and electron coupling capacity

The activity of individual mitochondrial electron transport chain Complexes was determined immediately after isolation of mitochondrial fraction [16], [66] from RVLM. Enzyme activity assay kits (MitoSciences, Eugene, OR, USA) for Complex I, II or IV, and ATP synthase enzyme activity microplate assay kit (MitoSciences) for Complex V were used in conjunction with a spectrophotometer (Thermo Fisher Scientific Inc.); the activity of Complex III was determined as described by Nadanaciva [67]. The activities of NCCR (marker enzyme for electron transport capacity between Complexes I and III) or SCCR (marker enzyme for electron transport capacity between Complexes II and III) were analyzed as reported previously [14]. Total protein in the mitochondrial fraction was estimated using a protein assay kit (Pierce, Rockford, IL, USA).

### Measurement of mitochondrial superoxide anion

Mitochondria-derived superoxide anion in RVLM was determined quantitatively by an ELISA method based on MitoSOX (Molecular Probes) [64] in conjunction with spectrofluorometer (Gemini EM Microplate Spectrofluorometer, Sunnyvale, CA, USA), or semi-quantitatively by the intensity of fluorescence determined by an analysis program (Fluoview FV10-ASW Version.02.01) in conjunction with laser scanning confocal microscopy (Olympus).

### Statistical Analysis

All values were expressed as mean ± SEM. The temporal effects of various treatments on MAP, HR or power density of the LF component were assessed using two-way analysis of variance (ANOVA) with repeated measures for group means. Individual biochemical parameters were assessed with one-way ANOVA. In both cases, Scheffé multiple-range test was used for post hoc comparison of individual means. Mortality was assessed with the Fisher Exact Test. *P<*0.05 was considered to be statistically significant.
